# Research Trends on Mobile Mental Health Application for General Population: A Scoping Review

**DOI:** 10.3390/ijerph18052459

**Published:** 2021-03-02

**Authors:** Won Ju Hwang, Ji Sun Ha, Mi Jeong Kim

**Affiliations:** 1 College of Nursing Science, Kyung Hee University, 26 Kyungheedae-ro, Dongdaemun-gu, Seoul 02447, Korea; 2 School of Architecture, Hanyang University, 222 Wangsimni-ro, Seongdong-gu, Seoul 04763, Korea; mijeongkim@hanyang.ac.kr

**Keywords:** mobile mental health, mobile app, m-health, stress management, mental health app

## Abstract

Background: Scoping reviews of the literature on the development and application of mental health apps based on theoretical suggestions are lacking. This study systematically examines studies on the effects and results of mental health mobile apps for the general adult population. Methods: Following PICOs (population, intervention, comparison, outcome, study design), a general form of scoping review was adopted. From January 2010 to December 2019, we selected the effects of mental health-related apps and intervention programs provided by mobile to the general adult population over the age of 18. Additionally, evaluation of methodological quality was assessed using the Scottish Intercollegiate Guidelines Network (SIGN) checklist. Results: Fourteen studies were analyzed of 1205 that were identified; duplicate and matching studies were excluded. One was a descriptive study and 13 were experimental, of which randomized control trials (RCTs) accounted for 71.4%. Four of the mobile apps were developed based on cognitive behavior theory, one based on stress theory, and one on ecological instant intervention theory. These apps included breathing training, meditation, and music therapy. Stress, depression, and anxiety decreased using these apps, and some were effective for well-being. Conclusion: With the rapid development of technology related to mental health, many mobile apps are developed, but apps based on theoretical knowledge and well-designed research are lacking. Further research and practices should be conducted to develop, test, and disseminate evidence-based mHealth for mental health promotion. RCT studies are needed to expand the application to mental health services to various populations.

## 1. Introduction

The demand to improve mental quality of life has increased along with the recent increase in interest in and awareness of mental health [1]. Consequently, the concept of mental health services emerged, along with an increased demand to manage mental health using information and communication technology (ICT) such as mobile communication and social network services (SNS) [2]. Mobile social media is a rapidly growing internet sector, in which, according to the Ministry of Science and ICT and the Ministry of the Interior and Safety, the number of smartphone users accounted for 94.0% of the total Korean population in 2017, with 86.8% of smartphone users using applications (apps). As such, the use of smartphones and apps have become a part of daily life. Recently, clinical use of mental health-related apps and intervention programs based on mobile apps have been implemented as an approach to managing mental health [3,4].

Mobile health (m-Health) is not yet well known in South Korea. The World Health Organization (WHO) defined mobile health (m-Health, mHealth) or mobile healthcare as medical and public health services provided through mobile devices including smartphones. In other words, mobile healthcare is the exchange of medical services between physicians and patients using IT and mobile devices, free from restrictions of time and space [5]. In that sense, the use of mobile apps for mental health management is expected to have various advantages [6]. In South Korea specifically, smartphone usage is approximately 90% and thus, interventions using readily available smartphones may grant excellent accessibility. It also offers economic advantages of reducing medical costs and can help overcome limitations of accessibility compared to the in-person cognitive behavioral therapy previously used for mental health management [7]. Furthermore, mobile apps enable user-friendly access through their various functions. According to FLURRY, a mobile app information analysis company, the use of health and fitness apps increased by more than 330% from 2014 to 2016 as smartphone usage became diversified—needless to say, apps have become an important tool in for pursuing a healthy lifestyle [8,9]. Mental health-related mobile apps are referred to as “Mental Health apps (MHapps),” and their types and numbers are diverse [10]. Nevertheless, mental health apps that have insufficient medical evidence or are for commercial use are common, and it is to be expected that such apps will continue to be developed and distributed in the future. Therefore, in order to efficiently manage the mental health of citizens, the development and distribution of apps with proven effectiveness and reliability are needed. Mental health specialists, in particular, should demonstrate leadership in studying, evaluating, and integrating such apps [3]. Nonetheless, there is a lack of scoping review identifying trends in research and evidence-based app development and application on topics such as the effects of mental health apps. Thus, this study aimed to perform a scoping review of previous research on the application of mental health apps on the general adult population and systematically analyze the content, methods, and effectiveness of the proposed intervention programs.

In this study, a scoping literature review was conducted on studies that examined the effectiveness of interventions that used mental health-related apps, using mobile devices such as smartphones or tablets, within the general adult population aged over 18 years, to outline the necessary implications for mental health practitioners and health promotion.

## 2. Methods

A scoping review of various databases was conducted, as outlined below, to identify intervention studies that used mental health apps for the general adult population over the age of 18, with the purpose of presenting directions and possible research questions for future research. It consists of six steps of identifying the research question, identifying relevant studies, study selection, charting the data, collecting, summarizing, and reporting results and recommendations [11,12].

### 2.1. Identifying the Research Question 

The focus of this study is reflected in the following research questions: “What trends have intervention studies followed when using mental health-related apps for the general adult population?” and “What is the effectiveness of interventions that use mental-health related apps?”

### 2.2. Identifying Relevant Studies

A literature search was conducted by 2 independent researchers from 1 October 2019 to 30 December 2019 on mental health app-related intervention articles published in a journal between January 2010 to 2019. For data collection, Korean search engines (RISS (Research Information Sharing Service) and DBpia) and international search engines (MEDLINE, CINAHL, EMBASE, PsycINFO, Cochrane Library) were used. Google Scholar was used to manually search and verify the references of selected articles for inclusion of grey literature. Language was limited to English and Korean.

The keywords used for searches using international search engines were: m-health or “mobile health;” “mobile app*”/or mms*/or smartphone*/or “ipad” or “ipod” or “iphone*”/or “ipod*”; mental health* or “mental health treatment” and “program*” stress* depress* or depression or exp “depressive disorder, general * anxi*” or “social anxi*”/exp anxiety disorders, well-being or wellbeing, emotional labor or emotional labor, resilience or resiliency or resilient. The keywords used for searches using Korean search engines were: mobile app, mobile application mobile*, smartphone, mental health, mobile health. The search strategy combined keywords using the Boolean operators “AND” and “OR,” and limited results to articles published in English or Korean. Furthermore, studies in which the subjects were not adults, studies in which subjects received psychiatric medication or treatment, studies without interventions using apps, studies without intervention outcomes, studies that did not provide original text, qualitative studies, review papers, and studies with irrelevant topics were excluded.

### 2.3. Study Selection

#### 2.3.1. Study Population

This study selected articles that targeted the general adult population aged over 18 years, thus excluding nonadult subjects, neuropsychiatric drug users or those undergoing treatment, studies without app interventions, and studies without research outcomes. Studies that did not involve original research, qualitative studies, review articles, and studies not related to the research area were also excluded.

#### 2.3.2. Interventions

The selected interventions were mental health apps that showed improvements through mobile use. This study was general in that all sorts of intervention methods that involved mobile use for the purpose of mental health promotion were selected.

#### 2.3.3. Comparisons

Comparative interventions included all intervention studies in which a comparison was made to nonintervention groups, waiting lists, and groups that received conventional treatment.

#### 2.3.4. Outcomes

The selected outcome was the effectiveness of the mental health app intervention, where studies that presented results of measurements using tools such as stress, depression, anxiety, and well-being were selected.

#### 2.3.5. Study Design

The selected study designs were randomized controlled trials (RCTs), nonrandomized controlled trials (NRCT), and research studies; 28 articles were selected based on the data selection criteria. The initial search by inputted keywords in respective databases yielded the following results: in the Korean literature, 37 articles in RISS and 59 articles in DBpia; in the international literature, 85 articles in MEDLINE, 344 articles in CINAHL, 493 articles in EMBASE, 116 articles in PsycINFO, and 54 articles in Cochrane Library; a total of 1205 articles.

**Figure 1:**
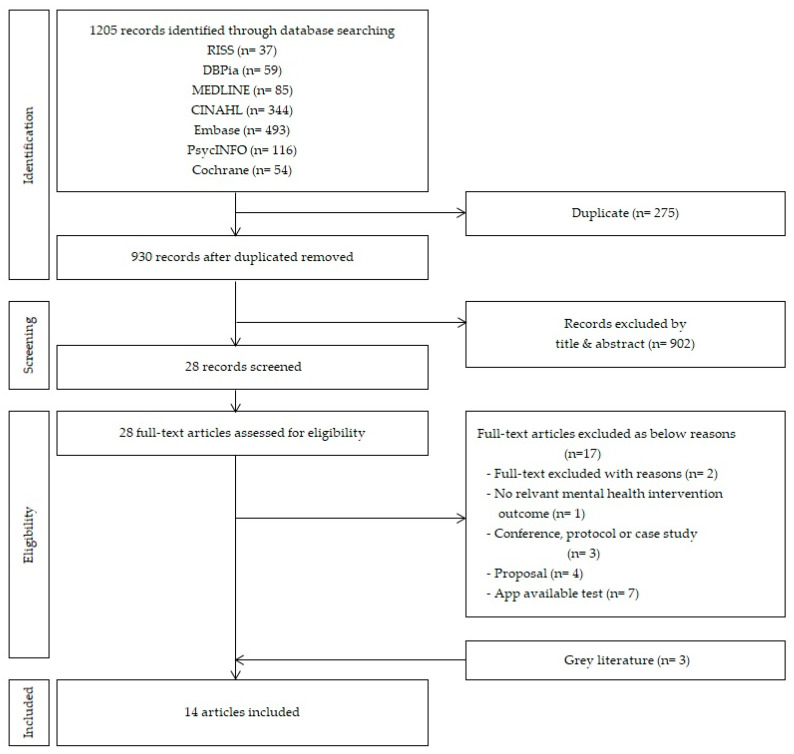
Flow diagram of the study selection processes for scoping review.

There were 258 articles that overlapped in the initial search, leaving 930 articles for review. Upon review of the titles and abstracts of the 930 articles, 28 articles met the selection criteria; 11 articles were selected upon further exclusion of studies based on app accessibility, studies that only presented preliminary research, study design, and case reports. Three articles were then added for inclusion of grey literature, based on a review of references used in major journals, consequently leading to a final selection of 14 articles for analysis (Figure 1). The literature search and selection process were independently reviewed by two researchers, and, in case of a disagreement, sufficient discussion on the reasons for article selection and exclusion was conducted.

### 2.4. Quality Evaluation of Selected Literature

To evaluate the quality of the selected literature, a critical review was conducted using the Scottish Intercollegiate Guidelines Network (SIGN) Checklist, a methodological quality assessment tool [13]. The SIGN Checklist involves a comprehensive evaluation of methodological quality of a study, a general evaluation of quality based on potential bias, and combined approach evaluating the type of study design and its execution [14]. The SIGN Checklists are comprised of the following 10 sections: appropriate and clear questions; randomization; adequate concealment; blinding; similarity of treatment and control group; treatment differentiation; standard, validity, and reliability of measurement devices; dropout rate; intention to treat analysis (ITT); and confidence between results at all sites. The primary evaluation consisted of “Yes” or “No,” “Cannot say,” and “Not applicable” as according to the checklist, while the overall quality of the studies were ranked based on the number of sections that received a “Yes,” for a final evaluation based on the following guidelines: studies with more than seven sections rated “Yes” were considered ++(high quality); studies with 5~6 ‘Yes’s were +acceptable; studies with 2~4 ‘Yes’s were –(low quality); and studies with less than one ‘Yes’ were unacceptable and therefore deleted. The quality evaluation process was independently conducted by two researchers; in case of a disagreement, the final decision was reached after consultation and sufficient discussion with a third researcher. 

### 2.5. Charting the Data

Two researchers analyzed and encoded the 14 selected articles. The encoded data were analyzed by serial number, researcher(year), name of the app, application program and theoretical evidence, study design, variables, number of samples, intervention subject, intervention period, and results.

Two researchers independently analyzed the 14 studies selected before December 2019, followed by a cross-analysis to ensure the appropriateness and accuracy of the content of the primary analysis. If discussions on omissions, errors, or inconsistencies were deemed necessary, the analysis was conducted after an agreement was reached following enough discussion. If there was any conflict, it was consulted to another author.

## 3. Results

### 3.1. General Characteristics of the Literature

As there were articles analyzed up until 2013 [15], articles from studies conducted after 2010 were included in the search. The general characteristics of the final 14 articles selected to analyze the effectiveness of mental health apps in adults, are as shown in Table 1. There was one article published between 2010 and 2015 (7.1%) [16] and 13 articles published after 2016 (92.9%) [17,18,19,20,21,22,23,24,25,26,27,28,29], which made up most of the selected papers. The study designs of the selected articles were one research study (7.1%) [17], 10 RCTs (Randomized Controlled Trial) (71.4%) [16,19,20,22,24,25,26,27,28,29], and three NRCTs (Nonrandomized Controlled Trial) (21.4%) [17,20,22]. In terms of the intervention subjects, there were various types, with three studies on normal adults (21.4%) [17,24,25], three studies on symptomatic cases (21.4%) [18,19,21], two workers (14.3%) [16,22], two nurses (14.3%) [23,29], one soldier (7.1%) [26], and three students (21.4%) [20,27,28]. The duration of intervention varied from 2 to 24 weeks, with nine studies less than four weeks long (64.3%), three 8-week studies (21.4%) [20,21,26], one 12-week study (7.1%) [25], and one 24-week study (7.1%) [22]. Over 60% of the selected studies lasted for less than four weeks [16,17,18,19,23,24,27,28,29].

### 3.2. Quality Evaluation of the Literature

Overall, the quality assessment of 13 of the selected articles, excluding one research study, yielded the following results: two studies [18,21] received a (-); one study [16] received a (+); and 10 studies [19,20,22,23,24,25,26,27,28,29] received a (++) (Table 2). The evaluation of the methodological quality of each of the selected studies indicated that all studies had adequately and clearly stated research questions [16,17,18,19,20,21,22,23,24,25,26,27,28,29]. Additionally, limitations in the quality assessment included issues regarding blinding and adequate concealment [19,26]), as well as cases in which a clear description of the survey procedure on app usage was not provided [16,29]. The dropout rate of study participants varied, from 0% [16,19,23,26] to 69.7% [25].

### 3.3. Summary of the Literature

One study [17] analyzed the effects of a mobile app (MoodPrism) through hierarchical regression analysis. MoodPrism is a self-monitoring app that examines the impact of subjects’ emotional self-awareness on their mental health. A program involving 234 participants demonstrated an impact of 18%, 20%, and 37% on depression, anxiety, and mental well-being, respectively (Table 3).

### 3.4. Experimental Research Using Mobile Apps

In total, there were 13 intervention studies using mobile apps [16,18,19,20,21,22,23,24,25,26,27,28,29]. The purposes of such mobile app interventions varied, including stress management, reduction of anxiety, reduction in depression, increase in well-being, and provision of feedback on mental health apps. The intervention period also varied, ranging from 2 to 24 weeks in duration. More specifically, there was one 2-week study [18], one 3-week study [16], three 30-day studies [17,20,24,28], two 4-week studies [19,23,27,29], two 8-week studies [21,26], one 12-week study [25], and one 24-week study [22].

For studies in which the purpose of the intervention was to provide feedback on the application of mental health apps, the results were measured in several ways. The result variables from the measurements of the effectiveness of the mobile app are as follows—13 articles on stress [16,17,18,20,21,22,23,24,25,26,27,28,29], nine articles on depression [17,18,21,24,25,26,27,28,29], 10 articles on anxiety [17,18,19,21,24,25,26,27,28,29], and five articles on well-being [17,20,24,27,29]. Furthermore, quality of life [19,20], self-efficacy [17,24,29], alcohol dependency [25], hyperventilation [19], sleep quality [27], work productivity [27], resilience [28], fatigue [23], mental health literacy [17,24], and emotional labor [29] were examined, and five articles [17,18,23,26,29] explored the accessibility and functional characteristics of the mobile applications (Table 3).

### 3.5. Mobile App Programs and Theoretical Evidence

The mobile apps used in the studies were, “It’s time to relax!” [16], “MoodPrism” [17], “ACT Daily” [18], “Flowy” [19], “Headspace” [20], “IntelliCare” [21], “internet-based stress management intervention (iSMI)” [22], “smartphone delivered mindfulness (SDM)” [23], “Mhapp (MoodKit, MoodPrism, MoodMission)” [24], “Cognitive Control App” [25], “mHealth” [26], “DeStressify” [27], “Headspace & Smiling Mind” [28], and “Mind Healer” [28]. Of the 14 articles, the most used apps were based on mindful meditation, used in five studies [16,20,23,27,28] (35.7%). Four studies used [23,24,25,26] cognitive behavioral therapy apps; one study [22] used a complex program involving problem-solving, relaxation, and acceptance tolerance therapy based on stress theory; one study [18] used an acceptance tolerance therapy app based on ecological momentary intervention (EMI); and another study [29] used a complex program involving meditation, yoga, and sound. There were two studies in which theoretical evidence was not provided, each using a breathing exercise app [19] and a mood-monitoring app [17].

- “It’s time to relax!” [16] is a stress management program developed by an Android smartphone app. Based on a mindfulness protocol, the app was designed so that users could follow instructions and practice meditation for free.
- “MoodPrism” [17] is an app developed to monitor and provide feedback on the user’s emotional state by converting the daily mood report to incorporate health aspects. The app also provides links on mental health information and resources.
- “ACT Daily” [18] is an EMI app designed to support the improvement and generalization of ACT (Acceptance and Commitment Therapy) technology, on which the users self-report emotions such as depression, anger, violent thoughts, and feelings of being trapped, and rate them on a Likert scale. Information is then provided on ways to alleviate such emotions.
- “Flowy” [19] is an app developed as a set of breathing-retraining exercises to manage anxiety, and uses simulations such as games to enable the user to subconsciously utilize breathing techniques.
- “Headspace” [20] and “smartphone delivered mindfulness (SDM)” [23] are based on mindful meditation techniques designed to encourage users to meditate for a few minutes each day to reduce stress, and provides information for a good night’s sleep.
- “IntelliCare” [21] is an app developed with the purpose of reducing depression or anxiety caused by sleep disorders, social isolation, or lack of physical activity, based on acceptance and commitment, conscious behavior, optimism, and problem-solving techniques.
- “iSMI (Internet-based stress management intervention)” [22] is an internet-based stress management program aimed to reduce stress, comprised of eight modules using problem-solving, relaxation, and acceptance tolerance therapy. Through adherence monitoring by an E-coach and presenting feedback pertaining to the user’s needs, an opportunity for the user to develop self-guided health promotion and behavioral change is provided.
- “Mhapp (MoodKit, MoodPrism, MoodMission)” [24] is a study involving the use of 3 apps—“MoodKit” and “MoodMission” are apps designed to manage depression, anxiety, and stress based on cognitive behavioral therapy. Upon analyzing mood, activity, values, and sentiment, it provides individualized goals that the user can choose and work towards.
- “Cognitive Control App” [25] is an app designed for the user to actively self-regulate behavior through choosing appropriate activities and refusing activities deemed inappropriate. It uses Cognitive Control Therapy (EVO), Problem-Solving Therapy App (iPST), Information Control (Health Tips) apps to facilitate both problem-solving abilities and provision of health information.
- “mHhealth” [26] is a mobile app with a combination approach involving wearables and cognitive behavioral therapy to reduce stress, depression, anxiety, and rage, developed to compensate for limitations observed in traditional approaches involving only cognitive behavioral therapy, such as dropout and loss, as well as the shortage of objective data between user experience and cognitive behavioral therapy sessions. Additionally, it provides objective data for the users and providers by identifying the user’s condition through cardiovascular and electrodermal input from wearable devices, enabling the detection of psychological stress.
- “DeStressify” [27] is a commercially available meditation app that provides guided meditation through audio, video, and text files. There are free and pro versions of the app, both of which are organized into visualization, gratitude, imagining one’s ideal life, and finding purpose. In the pro version, additional functions including the options “my friends,” “nutrition,” and “shop” are offered. Such functions are intended to manage symptoms of stress, anxiety, and depression.
- “Headspace & Smiling Mind” [28] is a preregistered meditation app with over 100,000 downloads on the Google play app with high-quality mobile ratings. ‘Headspace’ is designed for users to download the app and complete a basic 10-day training session on mindful breathing, body scan (systematically focusing on certain parts of the body), practice of nonjudgement of thoughts and emotions, and sitting meditation, then access other meditation tracks for the following 30 days using a prepaid voucher. “Smiling Mind” is a smartphone app developed by a psychologist and an educator that provides a variety of meditation programs for a diverse audience in different age groups. The adult program is designed for everyday use for 10 days, followed by continuous use for another 30 days to manage mental health using “Smiling Mind.” If the content is deemed insufficient, the user can select contents of their choice.
- “Mind Healer” [29] is an app developed for workers and the general adult population to manage stress and involves a psychological test and a PPG sensor that measures heart rate, enabling users to measure their mental health status, thus increasing workers’ self-awareness. Additionally, if stress, anxiety, or depression is detected, a short-term healing program is offered, providing breathing, meditation, music, and yoga practices for healing and management of mental health. By also providing materials for mental health education, the app enables users to promote mental health by themselves.

### 3.6. Effectiveness of Mental Health Interventions using Mobile Apps

The analysis of the 14 articles demonstrated that mobile mental health promotion apps were indeed effective in improving mental health. More specifically, mindful meditation apps [16,20] commonly demonstrated a significant reduction in stress, and when 56 Italian workers were subject to performing meditation for three weeks, significant reductions were observed in hyperactivity and accelerated behaviors in addition to stress [16]. Meanwhile, a 30-day trial of mindful meditation in 88 medical students also demonstrated a significant reduction in stress and increased well-being, displaying lasting effects of mindful meditation [20]. In a study involving a 4-week trial of mindful meditation in university students, a significant reduction in trait-anxiety and significant improvements in general health, energy, and emotional wellbeing were observed [27]. The use of an intervention app based on the stress model by 264 workers demonstrated a significant reduction in stress after seven weeks of use, which was maintained in follow-up observations from seven weeks to six months of use [22]. Furthermore, in a study involving the distribution of 626 normal adults into three groups, each using a Cognitive Control Therapy app, a Problem-solving Therapy app, and an Information Control app, no significant differences were observed between weeks 4 and 8, but a higher recovery rate was observed in the group using the Cognitive Control Therapy app at four weeks compared to the group using a health information app [25]. When adults with mental health disorders were subject to using apps that provided a 4-week training program on breathing, a significant improvement in quality of life was reported, along with reductions in anxiety, panic, and hyperventilation, though the observed differences were not significant [19]. In the case of the study on the IntelliCare app [21], in which interventions on commitment to acceptance, cognitive behavioral therapy, and positive psychology were provided to 96 adults, significant reductions were observed in both depression and anxiety. A 2-week intervention using an app providing EMI-based Acceptance and Commitment Therapy in 14 depressed and anxious patients demonstrated significant reductions in anxiety, psychological inflexibility, cognitive fusion, obstacle, and acceptance [18]. A study involving an 8-week provision of cognitive behavioral therapy through an app to 35 American soldiers demonstrated significant reductions in stress, anxiety, and depression [26], and when cognitive behavioral therapy was provided to 226 normal adults through an app, improvements in emotional well-being and self-efficacy in coping were observed, along with reduced anxiety. In the Moodkit and Moodmission groups, especially, significant reductions in depression were reported [24]. When cognitive control therapy was provided to normal adults with depressive symptoms for 12 weeks, significantly higher rates of recovery were observed after four weeks, compared to the control group [25]. When cognitive behavioral therapy was provided to 95 newly appointed pediatric nurses for 4 weeks, significant increases were observed in acting with awareness and nonreactivity to inner experience, along with reduced in burnout and increased compassion satisfaction. Additionally, in a 4-week trial of the Mind Healer app in workers, significant reductions were observed in stress and emotional labor, while significant improvements were observed in well-being and self-efficacy following app use [29].

## 4. Discussion

Given the recent emergence of mobile apps as a tool for mental health intervention, this study presents a scoping review of intervention studies that used mental health-related apps for the general adult population over 18 years, to present directions for future research.

The study identified intervention methods and their effectiveness, as well as implications for the further development and application of intervention programs for improving mental health in the general adult population, by analyzing intervention studies using mobile apps.

Mobile mental health application research has accelerated since 2016, which appears to be related to the rapid growth in the number of smartphone users [3,4]. Subsequently, the number of studies has increased as more people have access to mobile applications, and as the interest in and demand for mental health services has increased [1].

Experimental studies (92.8%) were the most used research design, of which well-designed RCTs accounted for 71.4%. On the contrary, in another systematic review of smartphone apps for treatment of mental disorders, it was found that RCTs are still a minority, at 15.8% [30]. In the future, a well-designed RCT study is needed to expand the application of mental health services to various populations and to present evidence for the effectiveness of these apps.

The participants in the reviewed studies were the general adult population, psychological clients, employees, students, nurses, and soldiers. Particularly, mobile mental health applications were applied as a primary preventive method for mental health management, not for patients diagnosed with mental illness but for the general population. In the future, studies that compare and analyze the trends of mobile application use and the primary preventive effects for general populations should be conducted.

Intervention studies on mobile apps involved the development of new apps as well as existing apps, depending on the purposes of the study. Approaches using wearables were also included to promote mental health. Nonetheless, real-life applications of such apps on the general adult population are currently limited, with most of the developed apps designed to target subjects who already have mental health problems or to support clinical treatments [29]. Additionally, screening apps designed to classify high-risk adult subjects in relation to stress, depression, and anxiety were being developed and operated [31]. It was demonstrated that various attempts on assessments and interventions using mobile apps were being made in a variety of aspects, with the majority targeting the general adult population [32]. As the stigma around psychological treatment is the biggest factor that hinders the mental health promotion, there is an urgent need for the use of mobile apps for the provision of intervention, education, and consultation services for mental health promotion in Korea [31]. Addressing mental health using mobile apps has the advantages of providing researchers with a database to make up-to-date observations and develop future interventions from, and providing users with a way to become self-aware of their current status and changes, considering most users must download and sign into the apps [33].

Although the duration of app use varied from two weeks to six months and the effectiveness of the apps were evaluated following the intervention in existing studies, there was a lack of research on the lasting effects of mobile app interventions. The analysis of the existing studies indicated a shortage of research on the prolonged use of the apps. Furthermore, Donker et al. [15] indicated that the rates of prolonged use of mHealth apps were low. Therefore, considering the sustainability aspect of the mental health promotion apps and the fact that rates of continuous use decrease with longer intervention periods, there is a need to identify ways that support the continuous use of the apps. As a response, there have been studies that have applied the information–motivation–behavioral skills model to mental health apps [31,34], which indicate that because motivation strengthens behavior [35], subjects who acquire both health information and motivation gain the capacity needed to continue to perform healthy behaviors, which brings about actual behavioral skills [36]. Therefore, it can be presumed that strategies involving active efforts to increase the effectiveness of interventions and sustainability will need to be inserted into the context of the app during development, provided through immediate motivation, or provided through continuous relevant feedback for emotional support in future mobile app applications [29]. On the other hand, it was difficult to compare the effectiveness of mobile apps as there were variables other than stress, depression, anxiety, and mental well-being measured to evaluate the effectiveness of the mobile app use and interventions, not to mention inconsistent results.

Recently, there has been a lot of meta-analysis of the effectiveness of mobile interventions in specific mental disorders and in health psychology [37,38,39]. Although meta-analysis was performed on the general population, the effects of mobile app intervention itself could not be confirmed because the effects of the interventions, mixed by mobile app and web-based interventions, were identified [40,41]. Meta-analysis of the effectiveness of mobile app intervention in the general population is still not enough. In the future, a meta-analysis on the effectiveness of mobile app interventions for the general population may be needed.

Upon analysis of the programs and theoretical evidence involved in mental health apps, it was evident that most mental health apps were developed based on therapy rather than a theoretical framework. There are more than 3000 mental health apps available for Android, Apple, and Microsoft [15,42]. In a recent review of the existing commercial mHealth apps for the most prevalent health conditions on the Global Burden of Disease list provided by the World Health Organization, it was concluded that the development of mHealth apps was driven by commercial and economic motives, rather than scientific motives, as observed by previous studies [43]. Therefore, there is a need for the development and distribution of mobile apps with evidence-based content [32], along with consumer education, to enable users to select reliable, effective high-quality apps [44].

Additionally, despite the large amount of mobile app development due to the rapid growth and development of mental health technology, there remain important issues and risks involving insufficient quality control [45]. As such, more research and processes for the development, testing, and distribution of evidence-based mHealth are needed to promote mental health effectively. Furthermore, there is a need for nationwide support in developing and distributing high-quality content for mental health management apps. Additional efforts are required to identify the best ways a mobile app can be used to address components [46] such as the development and application of methods to evaluate interactive mechanisms from physical measurements [47,48]. Mental health problems arise not only from congenital factors, but through a complex mechanism of various factors [49]. Thus, there is a need not only for customized apps that take the demands of the users into account, but also for mobile apps that enable the simultaneous management of physical and mental health alongside stress prevention [29,50].

Recently, there have not been many mental health intervention studies using mobile apps on the general adult population. Therefore, caution is required when generalizing and interpreting the results of this study. In the meantime, as the mobile app approaches and usage are increasing, mental health workers must continue to evaluate whether various apps are developed upon sound, scientific evidence [42,44,51] and whether the effectiveness of the interventions was examined using appropriate and reliable tools [32], and enforce reliability through further research [52]. Continuous management and attention may also be needed from the government to enable the use of efficient and effective mobile apps [53]. Additionally, efforts should be made to prevent health inequalities related to age, socioeconomic status, and health literacy by identifying and encouraging a wide range of smartphone and health app users [54]. The development and use of mobile apps taking such points into consideration will enable the effective and systematic management of adult mental health, and ultimately prevent and alleviate mental health problems.

This paper analyzed studies on apps used for mental health promotion in the general population to present a scoping review, which is a research methodology used to present future research directions to clinical practitioners and guide further studies. In addition, since the scoping review study does not perform quality assessments, there is a potential risk of methodological bias. To overcome methodological limitations, a quality assessment for practical studies was performed after selecting the literature.

This study included RCT, non-RCT, and descriptive studies for the literature review on the subject scope, and excluded theses and academic conference presentations, among other possible variants of presentational forms, because the analysis was focused on journal articles. Moreover, unpublished studies were not included in the analysis.

## 5. Conclusions

According to the results obtained in the study, there were a total of 14 studies pertaining to mobile healthcare service research done using mobile health promotion apps developed by mobile app providers on healthy adults to examine its effectiveness. The analysis of the literature demonstrated that mindful meditation was most applied in mental health intervention programs using mobile apps. Other intervention programs included cognitive behavioral therapy apps, complex programs made up of a variety of different components, and apps based on the stress model and breathing exercises. Mental health apps encouraged awareness of self and provided information pertaining to the user’s current status, and were comprised of components such as music, meditation, breathwork, quotes, videos, nature sounds, and health information. Such apps reduced stress, anxiety, and depression and improved well-being, but faced challenges in that there were only a small number of intervention studies, making the generalization of the study findings difficult. They may be helpful in the development and application of mobile apps for adults in the future.

Based on the results, the following suggestions can be made. A meta-analysis should be conducted on the research studies on mobile apps to confirm the effectiveness of apps. Moreover, the validity of mobile apps for mental health promotion for the general population can be improved by the development and application of mobile apps that satisfy the needs of users through further research on user demand.

## Figures and Tables

**Table 1:** General characteristics of selected 14 studies.

Variables	Categories	N (%)
Publication Year	2013∼2015 2016∼2019	1 (7.1) 13(92.9)
Research Designs	Randomized controlled trials Nonrandomized controlled trials Survey	10(71.4) 3(21.4) 1 (7.1)
Intervention Group	The general adult population Psychological clients Employees Students Nurses Soldiers	3(21.4) 3(21.4) 2(14.3) 3(21.4) 2(14.3) 1 (7.1)
Intervention Duration	below 4 weeks 8 weeks 12 weeks 24 weeks	9 (64.3) 3 (21.4) 1 (7.1) 1 (7.1)

**Table 2:** Quality evaluation of the selected studies.

Controlled Trial	Carissoli (2015) [16]	David (2018) [17]	Levin (2017) [18]	Pham (2016) [19]	Yang (2018) [20]	Mohr (2017) [21]	Ebert (2016) [22]	Wylde (2017) [23]	Bakker (2018) [24]	Arean (2016) [25]	Winslow (2016) [26]	Lee (2018) [27]	Flett (2019) [28]	Hwang (2019) [29]
1.1 The study addresses an appropriate and clearly focused question.	Y		Y	Y	Y	Y	Y	Y	Y	Y	Y	Y	Y	Y
1.2 The assignment of subjects to treatment groups is randomized.	Y		NA	Y	Y	NA	Y	NA	Y	Y	Y	Y	Y	Y
1.3 An adequate concealment method is used.	CS		NA	Y	Y	NA	Y	NA	Y	Y	N	Y	Y	CS
1.4 The design keeps subjects and investigators ‘blind’ about treatment allocation.	CS		NA	N	Y	NA	Y	NA	Y	Y	N	Y	Y	CS
1.5 The treatment and control groups are similar at the start of the trial	Y		*	Y	Y	NA	Y	Y	Y	Y	Y	Y	Y	Y
1.6 The only difference between groups is the treatment under investigation.	Y		NA	Y	Y	NA	Y	Y	Y	Y	Y	Y	Y	Y
1.7 All relevant outcomes are measured in a standard, valid, and reliable way.	N		Y	Y	Y	Y	Y	Y	Y	CS	Y	Y	Y	Y
1.8 What percentage of the individuals or clusters recruited into each treatment arm of the study dropped out before the study was completed?	0.0%		7.1%	0.0%	6.8%	5.7%	0.4%	0.0%	35.8%	55.4% (4 weeks) 64.3% (8 weeks) 69.7% (12 weeks)	0.0%	20.9%	1.0%	6.7%
1.9 All the subjects are analyzed in the groups to which they were randomly allocated (often referred to as intention to treat analysis).	Y		Y	Y	Y	Y	Y	Y	Y	Y	Y	Y	Y	Y
1.10 Where the study is carried out at more than one site, results are comparable for all sites.	CS		Y	Y	Y	Y	Y	Y	Y	Y	Y	Y	Y	Y
Overall assessment of the study.	+			++	++		++	++	++	++	++	++	++	++

Y = YES; N = No; CS = cannot say; NA = Not applied; * = intervention group.

**Table 3:** Characteristics and outcomes of selected 14 studies.

	Author (Year)	Name	Program/Evidence	Research Design	Intervention Group (N)	Period	Measure Tools	Results (Significant *)
1	Carissoli et al. (2015) [16]	“it’s time to relax!’’	Mindful apps, MindApps, both released from iTunes	Randomized Controlled Trial	Italian workers (56)	3 weeks	MSP, HR	Meditation group: improvement in coping with stress, reduction in hyperactivity and accelerated behaviors and heartbeats* Listened to music: improvement in coping with stress. Reduction in pain and physical problems and heartbeats *
2	Bakker and Rickard (2018) [17]	MoodPrism	MoodPrism	Survey	General adults (234)	30 days	PHQ-9, GAD-7, WEMWBS ESAS-R, MHLQ CSES, SDS	Reduction in depression * Reduction in anxiety * Increase in mental well-being *
3	Levin et al. (2017) [18]	ACT Daily	ecological momentary intervention, acceptance and commitment therapy(ACT)	Nonrandomized Controlled Trial	Depressed/ anxious clients (14)	2 weeks	DASS, AAQ-Ⅱ CFQ, VQ PHLMS, SUS	Reduction in depression * Reduction in anxiety * Reduction in overall psychological inflexibility * Reduction in cognitive fusion * Reduction in obstacles * Reduction in acceptance *
4	Pham, Khatib, Stansfeld, Fox, and Green (2016) [19]	Flowy	breathing exercises Diaphragmatic breathing	Randomized Controlled Pilot Trial	Adults with Common Mental Health Disorders (63)	4 weeks	eHEALS, GAD-7, OASIS, ASI-3, PDSS-SR, QLES-Q-SF Nijmegen Questionnaire	Reduction in anxiety, panic, hyperventilation Increase in quality of life *
5	Yang, Schamber, Meyer, and Gold (2018) [20]	Headspace	mindfulness	Randomized controlled trial	Medical students (88)	60 days	PSS, FFMQ GWBS	Reduction in perceived stress * Increase in well-being and sustainment *
6	Mohr et al. (2017) [21]	IntelliCare	acceptance commitment therapy, cognitive-behavioral therapy, positive psychology	Nonrandomized Controlled Trial	Adults With depressive /anxiety symptoms (96)	8 weeks	PHQ-9 GAD-7	Reduction in depression * Reduction in anxiety *
7	Ebert et al. (2016) [22]	iSMI (internet- based stress management intervention)	Lazarus’ transactional model problem-solving therapy, emotion regulation	Randomized controlled trial	Employees with stress symptoms (264)	6 months	PSS-10	Reduction (7 weeks) in stress and sustainment *
8	Morrison Wylde, Mahrer, Meyer, and Gold (2017), [23]	SDM (smartphone delivered mindfulness)	Cognitive Behavior Therapy	Nonrandomized Controlled Trial	Novice pediatric Nurse (95)	4 weeks	CFST LEC PCL-C FFMQ	Reduction in burnout Increase in compassion satisfaction Increase in “acting with awareness” and “nonreactivity to inner experience” *
9	Bakker, Kazantzis, Rickwood, and Rickard (2018) [24]	MHapp - MoodKit, - MoodPrism, - MoodMission	Cognitive Behavior Therapy	Randomized controlled trial	General adults (226)	30 days	PHQ-9, GAD-7 WEMWBS, ESAS-R, CSES MHLQ	Increase in mental well-being Increase in coping self-efficacy Reduction in depression (MoodKit and MoodMission group) * Improvement in anxiety *
10	Arean et al. (2016) [25]	EVO *iPST* Health Tips	EVO: Cognitive Control Therapy *iPST:* Problem-Solving Therapy Health Tips: Information Control	Randomized controlled trial	General adults with depressive symptoms (626)	12 weeks	PHQ-9, SDS GAD-7, IMPACT AUDIT-C	No difference at weeks 4 and 8 for the project EVO yielded higher rates of recovery at 4 weeks compared with the Health Tips group * Similar recovery between the iPST and Health Tips arms
11	Winslow et al. (2016) [26]	mHealth	Cognitive behavioral therapy	Randomized Controlled Trial	US military veterans (16)	8 weeks	SUDS, DASS PROMIS, TSST	Reduction in stress * Reduction in anxiety * Reduction in stress and depression (between time points)*
12	Lee and Jung (2018) [27]	DeStressify	Mindfulness	Randomized controlled trial	University students (163)	4 Weeks	PSS, STAI QIDS-SR, MDD PSQI, RAND WPAI	Reduction in trait anxiety * Improvement in general health, energy, emotional well-being *
13	Flett, Hayne, Riordan, Thompson, and Conner (2019) [28]	Headspace Smile Mind	Mindfulness	Randomized controlled trial	University students (208)	30 Days	CES-D HADS-A PSS, BRS FS, CAT CAMS-R	Improvement in depressive symptoms and resilience *
14	Hwang and Jo (2019) [29]	Mind Healer	Meditation, sound, yoga, health information	Randomized controlled trial	nurses (56)	4 Weeks	PSS-10, KOSS PHQ-9, GAD-10	Reduction in perceived stress and occupational stress * Reduction in emotional labor * Increase in self-efficacy, well-being *

* MSP: Measure du Stress Psychologique; HR: Heart Rate; PHQ-9: Patient Health Questionnaire-9; GAD-7: Generalized Anxiety Disorder 7-item scale; WEMWBS: Warwick-Edinburgh Mental Well-Being Scale; ESAS-R: Emotional Self-Awareness Scale- Revised; MHLQ: Mental Health Literacy Questionnaire; CSES: Coping Self-Efficacy Scale; SDS: Social Desirability Scale; DASS: Depression, Anxiety, and Stress Scale; AAQ-Ⅱ: Acceptance and Action Questionnaire; CFQ: Cognitive Fusion Questionnaire; VQ: Valuing Questionnaire; PHLMS: Philadelphia Mindfulness; SUS: System Usability Scale; eHEALS:. eHealth Literacy Scale; OASIS: Overall Anxiety Severity and Impairment Scale; ASI-3: Anxiety Sensitivity Index-3; PDSS-SR: Panic Disorder Severity Scale-Self Report; QLES-Q-SF: Quality of Life Enjoyment and Satisfaction Questionnaire-Short Form; PSS: Perceived Stress Scale: FFMQ: Five-Facet Mindfulness Questionnaire; GWBS: General Well-Being Schedule.* CFST: Compassion Fatigue Self-Test; LEC: Life Events Checklist; PCL-C: Posttraumatic stress disorder Checklist-Civilian; FFMQ: Five Facet Mindfulness Questionnaire; WEMWBS: Warwick-Edinburgh Mental Well-Being Scale; ESAS-R: Emotional Self-Awareness Scale- Revised; CSES: Coping Self-Efficacy Scale; MHLQ: Mental Health Literacy Questionnaire; SUDS: Subjective Units of Distress Scale; DASS: Depression, Anxiety, and Stress Scale; PROMIS: Patient-Reported Outcomes Measurement Information Scale; TSST: Trier Social Stress Test; PHQ-9: Patient Health Questionnaire 9–Item; SDS: Sheehan Disability Scale; GAD-7: Generalized Anxiety Disorder 7-item scale; IMPACT: Improving Mood-Promoting Access to Collaborative Treatment: AUDIT-C: Alcohol Use Disorders Identification Test.* PSS: Perceived Stress Scale; QIDS-SR: Quick Inventory of Depressive Symptomatology Self-Report; MDD: Major depressive disorder; PSQI: Pittsburg Sleep Quality Index; WPAI: Work Productivity and Activity Impairment; CES-D: Ceter for Epidemiological Studies Depression Scale; HADS-A: Hospital Anxiety and Depression Scale-Anxiety Subscale; BRS: Brief Resilience Scale; FS: Flourishing Scale; CAT: College Adjustment Test: CAMS-R: Cognitive Affective Mindfulness Scale-Revised; KOSS: Korean Occupational Stress Scale.
